# SODa: An Mn/Fe superoxide dismutase prediction and design server

**DOI:** 10.1186/1471-2105-9-257

**Published:** 2008-06-02

**Authors:** Jean Marc Kwasigroch, René Wintjens, Dimitri Gilis, Marianne Rooman

**Affiliations:** 1 Unité de Bioinformatique génomique et structurale, Université Libre de Bruxelles, CP 165/61, avenue Roosevelt 50, B-1050 Brussels, Belgium; 2 Service de Chimie générale, Université Libre de Bruxelles, CP 206/04, boulevard du Triomphe, B-1050 Brussels, Belgium

## Abstract

**Background:**

Superoxide dismutases (SODs) are ubiquitous metalloenzymes that play an important role in the defense of aerobic organisms against oxidative stress, by converting reactive oxygen species into nontoxic molecules. We focus here on the SOD family that uses Fe or Mn as cofactor.

**Results:**

The SODa webtool  predicts if a target sequence corresponds to an Fe/Mn SOD. If so, it predicts the metal ion specificity (Fe, Mn or cambialistic) and the oligomerization mode (dimer or tetramer) of the target. In addition, SODa proposes a list of residue substitutions likely to improve the predicted preferences for the metal cofactor and oligomerization mode. The method is based on residue fingerprints, consisting of residues conserved in SOD sequences or typical of SOD subgroups, and of interaction fingerprints, containing residue pairs that are in contact in SOD structures.

**Conclusion:**

SODa is shown to outperform and to be more discriminative than traditional techniques based on pairwise sequence alignments. Moreover, the fact that it proposes selected mutations makes it a valuable tool for rational protein design.

## Background

Normal cellular metabolism produces reactive oxygen species, whose accumulation is prevented by the action of SODs. These enzymes convert superoxide to hydrogen peroxide, which is then removed by glutathione peroxidase or catalase. However, overproduction of reactive oxygen species can occur in abnormal processes such as irradiation, aging and several diseases [1,2]. In such case, natural SODs may become insufficient to ensure detoxification. Therefore, a better understanding of how SOD function and the design of active SOD mimetics would be particularly important in view of treating the effects of oxidative damage [3] or using them as therapeutic target [4].

There are several forms of SOD enzymes that are generally classified according to their metal cofactor, *i.e. *Cu/Zn, Ni, Fe and Mn ions. We focus here on Fe and Mn SODs, which are prevalent in bacteria and mitochondria. The large majority of these SODs require specifically either Fe or Mn to perform their biological activity; only some, called cambialistic, function with both types of ions [5].

Fe and Mn SODs have very similar sequences and structures [6], and it is often quite difficult to distinguish the metal specificity on the basis of their primary, secondary or even tertiary structures [7,8]. Two groups of Fe/Mn SODs can moreover be defined on the basis of their oligomeric properties, as they form either homodimers or homotetramers in solution. This property too is quite difficult to detect on the basis of sequence and structure.

Consequently, the development of a prediction method that allows to tune the activity and specificity of SOD enzymes and to design specific SOD mutants is very challenging.

## Implementation

SODa is available trough the SODa home page (see Section Availability and requirements). The main program is implemented in C; the web interface and the processing of the results are performed using Perl, Bourne shell scripts, and PHP. The output files in PDF format are created with the "HTML To PDF" PHP class [9].

The SODa prediction method relies on datasets of annotated and aligned sequences and structures, from which residue and interaction fingerprints are derived [10,11]. These fingerprints form the basis of the SODa method: they are combined to predict if a target sequence is a SOD, its metal specificity and oligomerization mode.

### Datasets of aligned sequences and structures

The sequence dataset encompasses 374 SOD sequences, for which an assignment of the metal cofactor and oligomeric state is established on the basis of the SwissProt annotation [12] if available, and on literature resources otherwise [11]. It contains 234 dimers (116 Fe-specific, 102 Mn-specific, and 16 cambialistic SODs) and 140 tetramers (42 Fe-specific, 94 Mn-specific, and 4 cambialistic SODs). This set was used to derive SOD- and SODtype-fingerprints.

In addition to this sequence datasets, a structure dataset was considered, containing 17 high-resolution x-ray structures which were retrieved from the Protein Quaternary Structure server [13]. A list of these structures can be found in [11]. They were aligned using the SoFiSt algorithm [14] onto the *E. coli *SOD structure of Protein Data Bank code [15] 1isa [16]; this protein is chosen as representative SOD.

To obtain a global alignment of the 374 sequences of the learning set, each of them was aligned onto the 1isa sequence, using the CLUSTALX sequence alignment algorithm [17]. This alignment was manually improved on the basis of the structure alignment of the 17 SODs from the structure dataset [11].

### Derivation of SOD- and SODtype-fingerprints

Single residues and residue pair interactions that are conserved in all SOD enzymes (SOD-fingerprint) or are specific to a SOD type (SODtype-fingerprints) have been identified from a set of 374 aligned SOD sequences and 17 aligned structures, merging Fe- and Mn-specific SODs, dimers and tetramers (see above). Pair interactions are defined as residues whose side chain geometric centers are separated by 8 Å at most in one the 17 aligned SOD structures. These fingerprints form the kernel of the SODa method [11].

The SOD-fingerprint contains the residues and the residue pair interactions that are present in 80% at least of all 374 aligned SOD sequences, and are used to identify if a target sequence is a SOD or not. Among these, the four residues that bind the metal cofactor (His26, His73, Asp156, His160, following the numbering of the *E. coli *SOD 1isa [16]) are perfectly conserved, and so is Glu159 which makes a salt bridge with His160 across the dimer interface. The interactions in the SOD-fingerprint link the four central metal-bound residues to the residues situated in their immediate neighborhood on the first shell around these central residues. Several of these interactions occur across the dimer interface, which corresponds to the main channel leading to the active site.

The SODtype-fingerprints are used to predict what type of SOD the target sequence corresponds to, that is, whether it is an Fe dimer, Fe tetramer, Mn dimer or Mn tetramer. They contain residues and residue pair interactions that are typical of a SOD subgroup, *i.e. *which occur in at least 80% of the members of the subgroup and in less than 20% of the other SODs. In addition to the four basic subgroups (Fe dimer, Fe tetramer, Mn dimer, Mn tetramer), we also consider larger subgroups that are the union of several basic subgroups: Fe, Mn, dimers, tetramers, all but Fe dimers, all but Mn dimers, all but Fe tetramers, and all but Mn tetramers. As expected, the tetramer fingerprint involves residues in the region where the structure differs between dimers and tetramers. It has to be noted that dimer and tetramer fingerprints contain residues at different positions, whereas Mn and Fe specific fingerprints concern several identical positions occupied by different amino acids, which tune the preference towards Mn or Fe. Note also that the interaction fingerprints involve several π -π, cation-π, amino-π, H-bond and salt bridge interactions. The SOD- and SODtype-fingerprints are listed on the webpage (see Availability and requirements section).

### Prediction of SOD and SODtype

The SOD-fingerprint is used to perform the first prediction, that is, to identify whether or not a target sequence is an Fe/Mn SOD. For that purpose, the target sequence is aligned to a hidden Markov profile built from the 17 sequences of our structure set using the HMMER program [18] with the default parameters. On the basis of this alignment, the residues and interactions of the SOD-fingerprint that are conserved in the target are identified; note that an interaction is supposed to be present if the two residues forming the interaction occur in the sequence. The target is predicted to be a SOD if it contains all the perfectly conserved residues and interactions, and at least 40% of the others.

If the target sequence is predicted to be a SOD, the program goes over to the second prediction level, which consists of predicting whether it is an Fe dimer, Fe tetramer, Mn dimer or Mn tetramer. It uses for that purpose the SODtype-fingerprints. The target is assigned to the basic subgroup presenting the highest weight, evaluated as follows. If a residue or interaction specific of one of the four basic subgroups (*e.g. *Mn dimer) occurs in the target, a weight of 1 is added to the subgroup; if it is specific to the union of two basic subgroups (*e.g. *Mn), a weight of 1/2 is added to the two basic subgroups involved (in this case Mn dimer and Mn tetramer); if it is specific to the union of three basic subgroups (*e.g. *non Fe tetramer), a weight of 1/3 is added to the three basic subgroups involved (in this case, Fe dimer, Mn dimer and Mn tetramer). All the weights are normalized through division by the maximum possible weight of the subgroup, and expressed in percent. The subgroup with the highest normalized weight *w*_*max *_is the predicted one. If the next highest weight is larger than *w*_*max *_– 20%, the corresponding subgroup is predicted too. Thus, cambialistic SODs and non well defined oligomeric modes can be predicted by the SODa server.

To evaluate the performance of SODa, we compared it with the commonly used prediction method that assigns the metal cofactor and oligomer state on the basis of pairwise sequence comparisons.

### Pairwise sequence comparison method

In applying the pairwise sequence comparison method, four reference sequences are considered, which are representatives of the four SOD types, *i.e. *a dimeric Fe SOD (*E. coli*; accession number P0AGD3), a dimeric Mn SOD (*E. coli*; P00448), a tetrameric Fe SOD (*Streptomyces coelicolor*; Q9X469) and a tetrameric Mn SOD (human mitochondrial; P04179).

The target sequence is aligned onto these four reference sequences using the WATER program from the EMBOSS package [19]. The target is assigned the metal specificities and oligomer properties of the reference sequence that yields the best alignment score, defined as the WATER similarity score divided by the maximum score reached by aligning the reference sequence onto itself, and expressed in %.

## Results

### Assessment of the SODa predictions

To evaluate the predictions performed by SODa, we first applied it to all 374 SODs of the learning set. All these sequences but one were correctly recognized as Fe/Mn SODs, which amounts to a score of 99.7%. The prediction of their cofactor specificity and oligomer state reaches a score of 97%. To have an objective estimation of the predictive power of SODa, we compare it with the commonly used assignment method, where the target sequence is aligned onto SODs of each type and assigned the oligomer state and metal specificity of the most similar sequence, as described in Implementation. Clearly, the latter method yields less good results: the percentage of target sequences correctly assigned drops to 88%, with 30 incorrect assignments spread over all SOD subgroups. Moreover, the scores of the four subgroups are much more similar, which results in a drop of discriminating power [11].

As no cross validation was performed in the above predictions, the significance of their high scores could be questioned. To illustrate in more detail the power of the SODa prediction method, we applied it to predict 7 novel SOD proteins that were not present in the learning set used to design the program, and whose metal cofactor and oligomerization state were experimentally characterized. As shown in Table 1, the first 6 SODs have their type correctly predicted both by SODa and by the usual sequence comparison method. In contrast, the last sequence in the Table, the hyperthermophilic bacterial SOD of SwissProt id [12] a5pf69, is correctly predicted by SODa, but not by the sequence comparison method. Indeed, the latter method gives the same score of 19% for both the Mn and Fe tetramer SODs; the score is equal to 17% and 16% for Fe and Mn dimers, respectively. The four scores are very close, and it is thus impossible to make a reliable prediction of the SOD type preference with this method. The SODa scores are equal to 71%, 10%, 3% and 4% for Fe tetramers, Mn tetramers, Fe dimers and Mn dimers, respectively, allowing a quite better discrimination among SOD types.

**Table 1:** Comparison of the SODa predictions, the sequence comparison predictions, and the observed SOD types

Protein*	SODa prediction	Sequence comparison prediction	Observed
a0s5t8_hydat	Mn tetramer	Mn tetramer	Mn tetramer [22]
sodf_pseht	Fe dimer	Fe dimer	Fe dimer [23]
q314g3_desdg	Fe dimer	Fe dimer	Fe dimer [24]
q1h4d0_metfk	Mn dimer	Mn dimer	Mn dimer [25]
a3yg55_9gamm	Fe dimer	Fe dimer	Fe dimer [26]
a6vxc3_9gamm	Fe dimer	Fe dimer	Fe dimer [26]
a5pf69_9bact	Fe tetramer	Mn/Fe tetramer	Fe tetramer [27]

* SwissProt id [12]

### Mutations for rational SOD design

Another novelty and power of SODa lies in the list of suggested mutations that are likely to reinforce the SOD function, the specificity for the metal cofactor, or the dimeric or tetrameric character. We would like to emphasize that, for a set of experimentally characterized SOD mutations, the tendencies predicted by SODa have been shown to be in excellent agreement with the measured ones [11].

**Figure 1:**
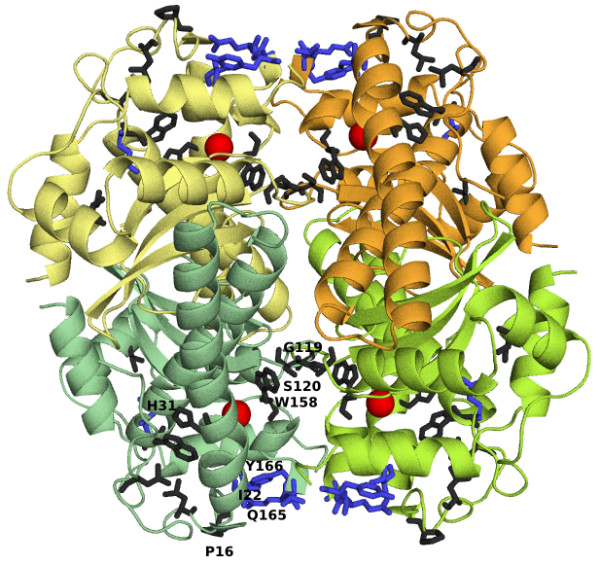
**Mutations predicted to enhance the Fe tetramer SOD characteristics of a5pf69**. The mutations are represented in the structure of PDB code 1ids [20], chosen as representative structure of Fe tetramers. Chains A, B, C and D are represented in green, limon, yellow and orange, respectively. The red spheres correspond to iron atoms. The mutations in a5pf69 that are predicted to reinforce the SOD characteristics are in dark grey (Leu7, Leu14, Pro16, His31, Trp77, Ile96, Gly119, Ser120, Trp158, using the 1isa [16] numbering) and those enhancing the tetramer mode are in blue (Ile22, Asn79, Leu164, Gln165, Tyr166). The residues that are discussed in the text are labeled. The figure has been generated using PyMol [21]

To illustrate the power of such mutations, let us return to the case of a5pf69 in Table 1. SODa proposes several mutations to increase its Fe tetramer SOD specificity. Among the substitutions likely to increase the SOD characteristics, let us mention the insertion of Gly119 (using the 1isa numbering), deleted in the a5pf69, combined with the mutations Tyr158Trp and Arg120Ser; these three residues interact across the dimer interface, as illustrated in Figure 1. Another proposed mutation is Tyr31His, a residue that is in contact with His26 in the reference structures, itself bound to the metal ion. This Tyr31His substitution moreover yields the characteristic His31-Gly69 interaction that is found in all but Fe dimers; note that Gly69 itself is typical of all but Fe dimers. Furthermore, the mutation Asp165Gln is assumed to increase the tetramer formation; this residue is in amino-π interaction with Tyr166 in the reference proteins, and interacts with the conserved SOD residue Pro16 and the tetramer-inducing residue Ile22, themselves substituted by Asn and Gln in the target.

## Conclusion

The prediction scores of the SODa method are higher and allow better discrimination between the four SOD types than the commonly used method based on pairwise sequence comparisons. This high discriminative power and the suggestion of targeted mutations makes the SODa server particularly well suited for rational design of SOD proteins, with modified or enhanced activity and specificity.

## Availability and requirements

SODa is freely available on the webpage .

The SODa user can submit a query by filling a form on the SODa web page. The sequence to be predicted must be in FASTA format. The "E-mail" field is required for later identification. The results are quickly (typically, after one minute) available on a web page, accessible via the link displayed or via the "results" page upon typing the E-mail address. The files remain available during seven days. The results of the prediction are described in three files named "align", "observed" and "missing", available both in HTML and PDF format.

The "align" file contains the main results of the prediction, in particular, whether the target is a "SOD" or a "non SOD" and, in the former case, what type of SOD it is. To allow the evaluation of the strength of the prediction, the percentage of residues and interactions from the target that match the SOD- and SODtype-fingerprints are indicated. Moreover, the alignment of the target sequence onto four reference proteins, one of each SOD type, is given. The residues and interactions corresponding to the SOD- and SODtype-fingerprints are colored in the alignment, and the missing characteristics are marked by an "X". Since it is impossible to indicate all the information in the alignment because some fingerprints overlap, the observed and missing characteristics are listed in the text files "observed" and "missing". The information in the latter file provides proposals for residue substitutions that are likely to reinforce the predicted SOD type.

## Authors' contributions

JMK implemented the cgi and the pre- and postprocessing tools; RW created the datasets, aligned the sequences, implemented the pairwise comparison prediction method; DG analyzed the mutants; MR derived the fingerprints and wrote the SODa prediction program.
